# Police violence and sexual risk among female and transvestite sex workers in Serbia: qualitative study

**DOI:** 10.1136/bmj.a811

**Published:** 2008-07-30

**Authors:** Tim Rhodes, Milena Simić, Sladjana Baroš, Lucy Platt, Bojan Žikić

**Affiliations:** 1 Centre for Research on Drugs and Health Behaviour, London School of Hygiene and Tropical Medicine, University of London, London WC1E 7HT; 2 Department of Ethnology and Anthropology, University of Belgrade, Serbia

## Abstract

**Objective** To explore female and transvestite sex workers’ perceptions of risk in the sex work environment in Serbia.

**Design** Qualitative interview study.

**Setting** Street based locations for sex work in Belgrade and Pancevo, Serbia.

**Participants** 31 female and transvestite sex workers.

**Results** Violence, including police violence, was reported as a primary concern in relation to risk. Violence was linked to unprotected sex and the reduced capacity for avoiding sexual risk. Participants reported that coerced sex was routinely provided to the police in exchange for freedom from detainment, arrest, or fine, and was enforced by the perceived threat of violence, sometimes realised. Accounts contained multiple instances of physical and sexual assault, presented as abuses of police authority, and described policing as a form of moral punishment. This was largely through non-physical means but was also enforced through physical violence, especially towards transvestite and Roma sex workers, whose experience of police violence was reported as relentless and brutal and connected with broader social forces of discrimination in this setting, especially towards Roma.

**Conclusion** Preventing violence towards sex workers, which can link with vulnerability to sexually transmitted infections, is a priority in Serbia. This requires monitoring perpetrators of violence, providing legal support to sex workers, and creating safer environments for sex work.

## Introduction

Physical violence links with vulnerability to sexually transmitted infections through coerced unprotected sex and the reduced capacity to negotiate sexual risk.^1^ ^2^ ^3^ Violence can be ubiquitous in street sex work.^4^ ^5^ ^6^ The incidence of physical violence, including rape, is higher among sex workers than among the general population.^7^ The emotional harms of sex work are often inseparable from the experience of violence.^6^ ^8^ ^9^ Violence links with social stigma and discrimination,^10^ ^11^ ^12^ and sex work is highly stigmatised.^5^ ^8^ ^11^

Preventing violence is a global public health and human rights priority.^13^ Policing and police violence can impact adversely on health.^14^ Street policing can displace sex work geographically as well as disrupt sex workers’ informal helping networks, exacerbating the risk of sexually transmitted infections as well as violence from clients.^11^ ^15^ ^16^ ^17^ Police contact with street populations can be pervasive and violent as well as stigmatising or discriminatory.^17^ ^18^ ^19^ ^20^ ^21^ ^22^ ^23^ Studies of injecting drug use suggest increased HIV risk behaviour linked to police contact and arrest.^24^ ^25^ ^26^ Excessive force, including physical and sexual violence, can be a feature of police “crackdowns” targeting street populations.^14^

Serbia is characterised by social and economic uncertainty, which links to poverty, illegal economies (including sex work), corruption, and ethnic and gender inequalities.^27^ ^28^ ^29^ Representations of violence linked to regional conflicts have been readily accessible through mass media, with uncertain long term consequences.^30^ There is a history of violence used as a stratagem for policing social unrest, and reports of police violence targeting vulnerable groups.^31^ ^32^ A culture of intolerance to social diversity and vulnerability has framed institutional responses.^29^ ^33^ Roma populations in Serbia and regionally are subject to pervasive inequalities in health, living conditions, citizenship rights, access to services, and income, and were subjected to violence in recent conflicts.^34^ ^35^ ^36^ In parts of eastern Europe most sex workers are thought to be Roma.^36^ We report findings from a qualitative study of the risk involved in a sex work environment in Serbia.

## Methods

We undertook 31 qualitative semistructured interviews with sex workers in two street based locations for sex work in Serbia; Belgrade, the capital, and Pancevo, a nearby town in Vojvodina region. The study included female and transvestite sex workers living or working in Belgrade or Pancevo who had exchanged sex for money, drugs, or other goods in the past month. This non-probability purposive sample set minimum quotas of outdoor and indoor sex workers, those under and over 21 years, injecting drug users, and Roma. We selected these purposive sampling dimensions to ensure variation in relation to contextual factors theorised to determine sexual risk differentially.^37^ Previous research highlights sex work environment, drug use status, and age as important determinants of risk related to sex work,^4^ ^5^ ^6^ ^18^ ^19^ ^20^ ^23^ and Roma are subject to noticeable inequalities in health, including risk of HIV and sexually transmitted infections.^35^ ^36^ We recruited participants through collaborating outreach projects at five discrete sex work “hotspots” and supplemented through snowballing and chain referral within networks of sex workers. We set an a priori target of 30 interviews with a fixed period for data collection.

We generated data using an interview topic guide containing key questions with prompts for patterns and organisation of sex work, use of condoms, risk perception, and risk in the sex work environment. The piloted topic guide was generated through close consultation with local sexual health and HIV prevention services, including non-governmental organisations, and fostered participant led description of experience related to sex work. Interviews were done in Serbian (by MS and SB) in cafes and restaurants (n=14), at local projects (n=12), at participants’ homes (n=4), and in quiet outdoor spaces (n=1). Participants were reimbursed €10 (£8; $16). Interviews were tape recorded after informed consent, lasted 60-90 minutes, and were transcribed verbatim by locally trained staff and the research team. Translated transcription was done through close collaboration with a translator, with translated text checked by bilingual members of the research team.

Coding of data was descriptive and thematic, with an emphasis on typological description.^37^ Data were collected in two waves to enable provisional coding to inform ongoing recruitment and purposive sampling as well as refinement of topic areas for interview. This enabled findings and codes to emerge iteratively.^37^ ^38^ At the interim analysis, violence (as defined by participants) emerged as a key theme and was thus given greater emphasis in later interviews. Coding of data was undertaken by TR and MS in two phases, with first level codes identifying key topics and concepts on the basis of a combination of participants’ accounts and a priori defined topics of interest (for example, violence), and second level codes breaking these into smaller units for analysis (for example, client violence, police violence, moral policing, non-physical violence).

The sample comprised 24 female sex workers and seven transvestite sex workers of average age 28 years. Most (n=25) were street based sex workers—that is, soliciting and negotiating sex with clients in street locations (beside busy roads, at railway and bus stations, at busy hotels). Street based sexual services were provided in nearby parking lots and woods, and in central Belgrade in street alleyways and rooms rented for 15 minutes. The remaining sex workers solicited sex through advertising their telephone numbers, and one was connected with a bar/club. Half were Roma (n=15), all of whom worked the streets, and among whom were all the transvestites. Two thirds of the women had children (n=15). Only five sex workers self reported sexually transmitted infections and eight reported injecting drugs, one of whom reported being HIV positive and another three hepatitis C positive. All street based sex workers reported having experienced some kind of violence linked to their work. Sex work is illegal in Serbia under article 14 of the Law of Peace and Order, and thus operates in a prohibitive environment.

## Results

Violence, and police violence in particular, was identified as a primary concern of sex workers in their descriptions of the risks involved in street based sex work in Belgrade and Pancevo, Serbia.

### Environment for sex work

As found elsewhere,^5^ ^6^ sex workers placed strong emphasis on condom use in transactional sex encounters: “Not using condoms with a client, God forbid!” (case 2, female); “With clients, it has to be with a rubber” (case 23, female). Condom use, as well as hygiene more broadly, was linked with personal integrity and responsibility: “I take good care of myself. All that [HIV] comes from the lack of hygiene, to those who don’t take good care” (case 8, female). A core strategy for regulating risk was “client sorting,” whereby the “messy,” “dirty,” and “dangerous” were avoided in favour of the “married,” “normal,” and “clean.” Judgments about hygiene influenced condom use, with unprotected sex said to occur discriminately and exceptionally under conditions of assumed low risk: “It happened, even without a condom, but he was married, clean, neat, spotless” (case 7, female). For some, economic necessity was a concomitant factor: “He paid more for the service” (case 7, female); “If he pays me extra, then I can do it without a condom” (case 6, transvestite).

Accounts thus emphasised the role of situational factors, and especially the risk of violence, in undermining a norm for condom use. The risk of violence seemed to be ubiquitous to this street setting for sex work, and was reported as a primary concern: “No kind of money is worth it when he beats you, when you are scared to death” (case 25, female). Violence was reported as linked to incidents during condom use or breakages resulting from rough or coerced sex: “If they push it in violently, then it [condom] breaks” (case 19, female). Violence was also described as being linked directly to unprotected sex through coercion: “I had to accept everything he asked for . . . without the rubber” (case 25 female). The perceived threat of physical violence also had indirect effects on sex workers’ capacity for reducing risk by feeding a sense of pervasive insecurity and loss of control in sex work transactions: “I get scared every time I get in [a car] with someone I don’t know, so that I am consumed with fear” (case 12, female); “I find it very difficult to get into someone’s car . . . because life is at stake” (case 22, transvestite). Physical violence by clients was reported to be common, but it was violence by police that was perceived as the greater threat and as less open to risk management: “You can manage your clients somehow, but to be honest, the greatest threat to us is the police” (case 7, female).

### Police violence

Although some participants had not experienced physical violence perpetrated by the police—“I perhaps got two slaps one time, that’s all, but other girls were hurt pretty bad” (case 16, female); “The police don’t know me well here, so I can stand freely where they don’t know me” (number 10 female)—the following forms of police violence seemed normative: deception and coercion (box 1), extortion (box 2), and discrimination (box 3).

**Box 1 Sex by deception and coercion**

**Extract 1: sex by deception**

And at the end of the job he shows me his badge, and says like “Give me my money back now.” That’s what he does. And he’s not on duty. But he’s some cop. *What do you do?* Give the money back (case 14, female)
He says “let’s do it.” I say, “Well, pay me, give me the money.” And then he asks, “Have you ever been taken in to the police?” Like he’s a cop and all. And I say, “Yes, I have.” And he says “Do you want to go to the station, you and I?” You know, they try you out, ask for blowjobs, sex in return. It’s just their thing (case 10, female)

**Extract 2: sex in exchange for freedom from police attention**

They want blowjobs, fucking, if you want them to let you go. You’ve got to. The police like fucking us more than anyone. They don’t pay. It’s like this: they fuck us, and so they let us go (case 5, transvestite)
And he pulled out a police badge and said “C’mon, you want me to take you in or screw you?” I was scared, and allowed him to screw me (case 18, female)
They want for blowjobs, fucks. I work for free, just so they don’t take me in (case 23, female)

**Extract 3: sex coerced by violence**

He wants me to blow him for free. I don’t want to. Later, when he gets me on my shift, he beats me silly. Beats me silly (case 20, transvestite)
He beat me up with a baton. Up in this park. He beat me up with the baton. And several times I had to be [have sex] with him. I really had to. I was forced (case 15, female)

**Box 2 Extortion**

**Extract 1: extortion of money**

They are screwing around with us. Like we need fuel, we need money for this, we need it for that (case 2, female)
Yesterday, or the day before, the patrol, they wanted money from us. First he says, “C’mon, get in the car.” Nothing to it, I get in the car. Then he says, “Why don’t you treat us?” And he says, “C’mon, sort it out among yourselves,” how much money we are to give him (case 14, female)
It’s really pathetic taking money from us. I don’t know how they don’t understand I struggled for that. I sold my body. I worked. The man, for instance, pardon me, fucked me and everything, for the money. And they take the money. Why? I don’t know, but so they say it goes into some fund, what do I know? (case 16, female)

**Extract 2: extortion of information**

The first time they beat me because I didn’t want to admit who I worked for. They slapped me around. Like, “Gypsy, motherfucker, why don’t you start talking?” I was pregnant, and he started beating me. Like, “You ain’t going to say you’re pregnant any more, now you’re going to get beaten up at the station, and nobody’s going to believe you, and if you report me, I beat you up” (case 8, female)

**Box 3 Discrimination**

**Extract 1: moral punishment**

They [police] take us into their office, and one starts kicking you in the legs, the other one into kidney. Without any reason. They want to accomplish something, to prevent us from doing something. *From what?* From doing this work. “Why don’t you find another job?” I say, “Come on, find me another job and I will do it” And he goes, “Why should I look for a job for you?” and so on. It’s like that (case 2, female)
He [policemen] is swearing at us, saying we’re sluts, whores . . . Pancevo is forbidden grounds to us. We can’t even move on the streets, nor in bars. If I had a boyfriend now, and went for a drink, and he came, he’d kick me out of the place (case 3, female)

**Extract 2: public humiliation and shaming**

A few times I went out with my boyfriend. I would sit down, drink coffee, and they [police] come in. “Hey, do you know . . .?,” I mean, it’s the first thing they say, “Do you know what she . . .?” And he [my boyfriend] goes, “Well, can I not have a drink with her?” “You know, well . . . Do you know what, do you know what she does?” (case 15, female)
Most of the women get arrested. The last time they arrested people it was on television. I saw it in person. Arrested, chased, and filmed on camera (case 1, transvestite)

**Extract 3: extreme violence driven by contempt**

They [police] started going wild, only on us transvestites. They let the girls go. They just pick us up, and go to the woods, and go wild on us . . . First, they beat us in the woods, and then they take us to the station. And then they tell us at the station “Hey, freshen up,” and they beat us up in the bathroom (case 5, transvestite)
They [police] kicked, kicked, kicked the hell out of us. Just transvestites. They took me to the woods, down by the bridge . . . They stripped everything off me . . . Flashlight in the eyes. I said a million times, “Take me away. Did you come to arrest me? Arrest me then, but, do not beat me up.” . . . That makes it worse. “Shut up, motherfucker, shut up!” (case 20, transvestite)
What’s the reason for them [police] to beat me up? The fact I’m like this is my business. One squad came over, and they beat me senseless because of what I am. I came back all black and blue . . . I didn’t know where the blows were coming from . . . They just have this hate. Whether it’s towards prostitutes or specifically trannies. But it’s terrible (case 22, transvestite)

Sexual services were described as being commonly provided to police without payment as well as secured by them through deception and coercion (box 1). Police reportedly presented sex workers with the option of providing services without payment as a means of avoiding possible arrest, detainment (varying from hours to one month), and a certain fine (in Belgrade, between €100 and €200). Sex workers described how in cases of deception, presumed clients would usually reveal their identity as policemen once services had been negotiated and exchanged, demanding the return of their money (box 1, extract 1). This was described as a no choice situation, which for some had become routine: “They’re in power, and we are not, what can I do?” (case 2, female). Although exceptions were reported (“Let me tell you, I didn’t work with none of them police”; “I don’t do cops”) (case 3, female),sex would normatively be provided in exchange for freedom from detainment or arrest (box 1, extract 2).

The risk of detainment, arrest, or fine or the threat of physical violence were given as the reasons whysex workers acquiesced to police demands for sex without payment. Attempts to resist such demands were said to incite violence (box 1, extract 3). We were given multiple examples of coerced sex involving violence perpetrated by those presumed to be police (box 1, extract 3), although condoms were reported to be used in most: “Leave off mate, not only am I doing you for free, but you’re not jacking me up without a rubber” (case 1, transvestite). Accounts also indicated routine extortion of money from sex workers by police, as unofficial fines or as “negotiated” payments to secure freedom from detainment, arrest, or registration as a prostitute (box 2, extract 1). Extortion of money or information (particularly about pimps and when giving “statements by dictation”) was also reported to be enforced by the threat of violence (box 2, extract 2).

A common theme therefore was a fatalist acceptance to the inevitability of everyday police violence (“I can’t fight destiny”), borne out of the internalisation of police “rights” to victimise (“They have a right to beat us because we do this prostitution thing”) and recognition that rights to police protection are unrealised and unrealistic (“I can’t complain to anybody, I’m a prostitute”).

A striking feature of sex workers’ accounts was that police violence was presented as transgressing boundaries of legal acceptability or rationality, and thus was thought to be moral punishment (box 3, extract 1). Enforced sex and coerced payments to police, although outside the law, were experienced as discipline as if for moral wrongdoing, to “bring sex workers to their senses,” and suggesting that they were matter out of place (box 3, extract 1). Policing thus extended into private life. A common tactic reported to be deployed was humiliation in the face of “normal others.” This involved unwanted disclosure of a sex worker’s identity to friends and family, thus disrupting the preservation of a dignified private self away from work (box 3, extract 2). A striking instance of public shaming included police collaborations with national media, whereby police crackdowns and arrests of sex workers would be televised (box 3, extract 2).

Moral punishments for selling sex overlapped with other forms of social discrimination, especially towards Roma and transvestites: “There is nothing [about me] that is normal for our people here, for our nation, here in Serbia” (case 1, transvestite). Police violence was described as especially brutal towards transvestites, all of whom in this study were Roma, and most Kosovo refugees (box 3, extract 3). Extreme violence reported towards transvestites and Roma was generally interpreted as driven by contempt for being of minority or deviant status (box 3, extract 3).

Taken together there was strong emphasis in sex workers’ accounts on the need for greater protection from police violence and corruption as well as for creating safer and regulated off-street environments for sex work, including through legislative change: “Legalise it. I’ll be the first to pay taxes” (case 2, female); “Make it legal, so that we have some papers and do not get messed with by the police” (case 8, female); “[Legalising] it would bring a proper solution. I could work freely. I would be up for HIV testing. I’m here to pay taxes. I’m here to fulfil my obligations to the state” (case 1, transvestite); “We need some building—I’m standing in the street—so that the police can’t touch us, some workplace” (case 12, female); “If it was legal, we’d go to doctors, we’d have medical records, it’d be much better” (case 15, female).

## Discussion

Violence was reported as a primary risk concern of street based sex workers in Serbia and linked to coerced unprotected sex, breakages of condoms, and reduced capacity to negotiate sexual safety. Moreover, sex workers described being coerced into providing sex to police in exchange for freedom from detainment, arrest, or fine, and this was reportedly enforced by perceived risks, sometimes realised, of physical violence by police. Reports of police violence, deception, coercion, and extortion towards street sex workers in Serbia seem to contravene the Serbian police ethics guidelines, ratified in 2003 and based on the European Union Code of Ethics for Police Forces (2001). 

This study has limitations. It is an exploratory study generating findings for testing in future research. Generalisability is inevitably limited to this specific sample and setting. The study is an interpretative analysis of participants’ accounts and reflects the themes of these. Counter narratives are possible, and the study did not investigate accounts by the police. Also, what distinguishes excessive force from legitimate force in policing can be a fraught debate.^39^

Interviews contained multiple descriptions of physical and sexual assault, presented as abuses of police authority. Reported coerced sex by police usually involved condoms, but the pervasive risk of violence contributed to a reduced sense of control over the negotiation of sexual transactions. Sex workers largely adopted a fatalistic acceptance to reported police demands for money or sex without payment. This study extends concepts of sex in exchange for money or goods to include coerced sex in exchange for freedom from police detainment, arrest, or fine.

This study also identified possible policing practices with little basis in legal rationality but which acted as forms of moral punishment. These practices discipline and stigmatise, positioning sex workers as having waived rights to respect or protection on account of having transgressed. Such practices usually comprised bullying of a non-physical nature, such as humiliation and shaming involving the unwanted disclosure of a sex worker’s identity to “normal others.” But discriminatory policing practices were also embedded in physical violence, and much of it brutal, as in the case of the relentless violence reported by transvestite sex workers, all of whom were Roma. Previous studies have noted excessive use of force when policing female or transvestite sex workers^17^ ^20^ and drug users.^14^ ^40^

Police violence may be disproportionately directed towards vulnerable groups.^39^ ^41^ It is shaped by organisational culture as well as social context.^41^ ^42^ ^43^ Violence linked to sex work reflects institutionalised social inequalities and anxieties relating to gender, ethnicity, sexuality, and vulnerability.^12^ ^44^ In Serbia, Roma are subject to immense social discrimination and impoverishment,^33^ and we found that Roma transvestite sex workers reported being subjected to extreme acts of police violence.

In conclusion, physical violence, enacted or threatened, links with unprotected sex and vulnerability to sexually transmitted infections^1^ ^2^ ^3^ ^4^ ^5^ ^20^ and can contribute to sex workers’ diminished sense of volitional control.^10^ ^11^ ^12^ ^17^ The prevention of violence tends to focus on reducing violence by clients, including through negotiation strategies of transactions.^8^ ^23^ A need exists to “design out” violence from sex work.^8^ This requires interventions to monitor client and police perpetrators of violence and contraventions in police conduct, legal support protecting sex workers’ health and human rights, and the creation of safer environments for sex work. The criminalisation of sex work environments exacerbates the vulnerability of sex workers to violence by police and clients while limiting sex workers’ access to protection.^18^ ^23^ ^45^ Interventions creating licensed regulated premises for sex work and enabling legislative frameworks protective of sex workers’ health, safety, and labour rights should be considered.

**What is already known on this topic**

Violence can link with coerced unprotected sex and risk of sexually transmitted infections, but little is known about police violence towards female and transvestite sex workers

**What this study adds**

In Serbia, coerced sex was reported to be routinely provided to police in exchange for freedom from detainment, arrest, or fine, and was enforced by the perceived threat of violence, sometimes realised
Policing acted as moral punishment through non-physical means but was also reportedly enforced through physical violence, especially towards transvestite and Roma sex workers

## Notes

Ethical approval: Riverside ethics committee (UK), with additional support from the Republican AIDS Commission of Serbia.

Provenance and peer review: Not commissioned; externally peer reviewed.

## Notes

**Cite this as:** *BMJ* 2008;337:a811
